# PI3K/AKT signaling mediate collagen type 1-induced osteogenic differentiation of dental pulp stem cells via focal adhesion mechanism

**DOI:** 10.1590/1678-7757-2025-0173

**Published:** 2025-08-18

**Authors:** Nur Julia Nabila NASIR, Norsyahida ARIFIN, Khairul Bariah AHMAD AMIN NOORDIN, Norhayati YUSOP

**Affiliations:** 1 Universiti Sains Malaysia School of Dental Sciences Basic and Medical Sciences Department Kubang Kerian Malaysia Universiti Sains Malaysia, School of Dental Sciences, Basic and Medical Sciences Department, Kubang Kerian, Malaysia.; 2 Universiti Sains Malaysia Institute for Research in Molecular Medicine Penang Malaysia Universiti Sains Malaysia, Institute for Research in Molecular Medicine, Penang, Malaysia.

**Keywords:** Bone, Scaffold, Stem cell, Signaling pathway, Osteogenesis, Tissue engineering

## Abstract

Dental pulp stem cells (DPSCs) are widely available sources of stem cells that have been extensively studied for its capacity to differentiate into osteoblasts and endothelial cells and to support bone repair and regeneration. Collagen type 1 (Col-1) is a well-known extracellular matrix component, which plays a vital role in regulating the signaling pathway for osteoinduction of bone progenitor cells. However, the exact mechanism of Col-1 activation during stem cell osteogenesis remains unclear.

**Objectives:**

This study aims to identify the key signalling pathway and proteins interaction associated with Col-1-induced osteogenesis of DPSCs.

**Methodology:**

The localization of OCN protein was assessed by immunocytochemistry analysis, followed by Western blot analysis on OCN, AKT, p- AKT, Smad2/3, p-Smad2/3, ERK1/2, and p-ERK1/2 pathways. Protein profiling was performed using gel-free digestion and LC-MS/MS, followed by protein-protein interaction analysis using STRING online tools to assist in determination of link between various pathways.

**Results:**

The data indicated that the PI3K/AKT pathway is the key signaling pathway involved in Col-1-induced DPSC, showing a significant impact and potential crosstalk with TGF-b/Smad and MAPK/ERK mainly via focal adhesion protein complexes.

**Conclusion:**

The evidence suggests that PI3K/AKT signaling pathway is more dominant than the TGF-β/Smad and MAPK/ERK pathways, acting via stimulation of the focal adhesion protein complex. Together, these findings may provide deeper insight into cellular biology of differentiated cells for potential manipulation in bone tissue repair and regeneration.

## Introduction

Bone structures serve fundamental functions in the body by protecting organs responsible for speech, smell, mastication, breathing, and hearing. Bone injury and defects are often associated with long-term physiological, functional, and aesthetic impacts, which can cause facial disfigurement and loss of mechanical function.^1,2^ Bone reconstruction seeks to restore the functionality and aesthetic structure of the deficient bone sections by providing structural stability and improving facial appearance.^3^ However, challenges exist in establishing an ideal condition to design and develop scaffolds appropriate to the needs of regulated bone regeneration.^4^

The traditional procedure of bone grafting has significant drawbacks, including limited graft tissue and disease transmission hazards. Cell-based tissue engineering seeks to regenerate new bone tissues by optimizing the three primary components, namely biomaterials, cells, and growth factors, in the defective areas.^5,6^ The interaction of these components in an optimal environment, together with proper nutrients and growth circumstances, enables cells to develop and differentiate into the desired tissue. Mesenchymal stem cells specifically isolated from dental pulp tissue are a promising source for cell-based therapy due to the non-invasive purification procedures, long-term proliferative properties, and multilineage differentiation capability.^7^

Collagen type 1 (Col-1) scaffolds mimic the natural extracellular matrix (ECM) environment, promoting cell-matrix binding to facilitate cell adhesion and spreading.^8^The incorporation of Col-1 in bone scaffolds acts as a physical support for progenitor cells to attach and proliferate by influencing the cell behavior and outcome via receptor-mediated interactions.^9^ Despite the increasing number of *in-vitro* studies performed on stem cells and Col-1, a clear understanding of the key mechanism involved in Col-1-induced osteogenesis of DPSCs and the associated microenvironment is still lacking.

The capability to comprehend the predominant signaling pathway in osteogenic differentiation induced by an optimized scaffold will offer a better opportunity to promote bone tissue regeneration. However, most of the studies on dental stem cells only focus on their potential in tissue engineering, without in-depth understanding of the direct impact of scaffolds and the activation of key signaling pathways involved. Moreover, the stimulation of the signaling pathway in cells is commonly not sufficient to assess its significance, as protein modifications of many types can impact the functioning of the protein in the cell.

Therefore, this study aims to elucidate the predominantly activated signaling pathway induced by Col-1 during the osteogenic differentiation of DPSCs by analyzing the key activated proteins during the process. Further proteomics analysis also provides detailed information on protein-protein interaction, downstream targets, and inhibitory networks, as well as the kinetics of osteogenic activation. The findings gathered from this study may then be used to manipulate internal or external factors to stimulate the outcome of Col-1-induced, cell-based bone regeneration by targeting the key pathway in osteogenesis for bone regenerative therapy.

## Methodology

### Fabrication of Collagen type 1 (Col-1) scaffold

A total of 2 mg/ml of rat tail Col-1 (Thermo Fisher, USA) was prepared following the manufacturer’s instruction. The mixture of collagen, 10X PBS, 1N NaOH, and distilled water were mixed in a tube and dispensed into culture plates. The plates were then incubated at 37℃ in a humidified incubator for 40 minutes before seeding.

### Cell culture

DPSC (#PT-5025, Lonza, USA) from passage 6-8 was employed in all experimental work in this study. DPSC cultured in alpha Minimum Essential Medium (α-MEM, Thermo Fisher Scientific, USA) with 10% fetal bovine serum (FBS, Thermo Fisher Scientific, USA) and supplemented with 1% penicillin/streptomycin (Thermo Fisher Scientific, USA). The cells were observed and incubated at 5% CO_2_, in which the medium was changed every three days. The cells were passaged when reaching 70% confluency.

### Osteogenic differentiation assay

DPSC were plated at 5000 cells/cm^2^ in 24-well collagen-coated plates. After reaching 80% confluency, the culture medium was replaced with osteogenic induction medium and incubated for 21 days. The medium was changed every three days. After 21 days, DPSCs were fixed with 10% paraformaldehyde (PFA) for 15 minutes, followed by staining with 2% Alizarin red S for 30 minutes. Finally, the cells were washed with 1X PBS (Gibco, USA) and observed under an inverted microscopy.

### Immunocytochemistry analysis

Cells were seeded at 5000 cells/cm^2^ in 8-well collagen-coated chamber slides (SPL Life Science, Korea) along with pathway inhibitors supplementation (LY294002, LY3200882, and PD98059). At 21 days of osteoinduction, the cells were fixed with 4% PFA for 15 minutes, and rinsed thrice with 1X TBS. Blocking was performed using 1% bovine serum albumin (BSA, Sigma-Aldrich, USA) for 1 hour, then washed with 1X TBS. Subsequently, the cells were incubated with primary antibody, Osteocalcin (1:100, Santa-Cruz) for 1 hour at room temperature. The secondary antibody, Alexa Fluor 688 (1:1000, Thermo Fisher Scientific, USA) and phalloidin FITC (1:1000, Abcam) were added for another 1 hour at room temperature in the dark. After washing, the cells were mounted with DAPI mounting medium (Gibco, USA).

### Pathway inhibition assay

Col-1 coated plates were seeded with DPSC at 5000 cells/cm^2^. Then 50 μM of PI3K/AKT inhibitor (LY294002), 50 μM of TGF-β/Smad inhibitor (LY3200882) and 50 μM of MAPK/ERK inhibitor (PD98059) were added into serum-starved cells at 24 hours and 15 minutes before protein extraction.

### Protein extraction and quantification

Protein was extracted at day 7, 14, and 21 using RIPA lysis and extraction buffer (Thermo Fisher Scientific, USA) along with the protease inhibitor cocktail I (R&D Systems) and Halt^TM^ phosphatase inhibitor cocktail (Thermo Fisher Scientific, USA) with the ratio of inhibitor to lysis buffer at 1:100. Protein concentration was then determined using the Pierce^TM^Bicinchoninic acid (BCA) assay kit (Thermo Fisher Scientific, USA).

### Protein separation

In total, 20 ug of protein lysates was added into 20μl of 1X Laemmli SDS-PAGE buffer and boiled at 95℃ for 5 minutes and centrifuged for 2 minutes at 9000 rpm. A total of 10 μl of protein samples and 10 μl of Precision Plus protein dual color standards (Bio-Rad, CA, USA) were loaded into a 10% Mini-PROTEAN TGX Precast Gel (Bio-Rad, CA, USA) and run at 150 V for 57 minutes. All gels were imaged using the stain-free application on the ChemiDoc MP (Bio-Rad) imager immediately after protein separation.

### Western blot transfer and chemiluminescent detection

The protein gels were blotted using the Trans-Blot Turbo transfer apparatus (BioRad, USA) for 7 minutes at 25 V. After transferring, the blot was incubated for 1 hour in a blocking buffer consisting of 1% BSA (Sigma-Aldrich, USA) in Tris-buffered saline containing 0.1% Tween-20 (TBST). The blot was then probed with primary antibodies at 4℃ overnight; osteocalcin D-11 (1:1000, Santa-cruz Biotechnology), pan-AKT (1:1000), phospho-AKT S473 (1:500), phospho-AKT T308 (1:500); phospho-Smad2/3 (1:1000), total ERK1/2 (1:1000), phospho-ERK1/2 (1:1000) (USA), and total Smad2/3 (1:1000, Novus Biologicals, USA), followed by 1 hour incubation with HRP-conjugated anti-mouse or HRP-conjugated goat anti-rabbit secondary antibody (1:1000, R&D Systems, USA). β-actin was included as the loading control (1:1000, R&D Systems, USA). Clarity western ECL substrate chemiluminescent detection reagent (Bio-Rad) was added and incubated for 5 min prior to image acquisition.

### Blot imaging and densitometry

The chemiluminescent blots were captured using the Chemi with Visible Marker protocol by FluorChem M Imaging System (Bio-Techne, USA). The captured images were then subjected to densitometry analysis using the Gel Analyzer tool of ImageJ to determine the profiles of each lane of the gels.^47^

### Statistical analysis

The immunoblot band data were expressed as mean ± standard deviation. The assumption of normality was assessed using the Shapiro–Wilk test, and all datasets were confirmed to follow a normal distribution (p>0.05), justifying the use of parametric tests. Statistical analysis was conducted using two-way ANOVA to evaluate the effects of treatment and time (days), including their interaction. The ANOVA results included F-values=3.5, degrees of freedom (2), and associated p-values<0.05. Significant differences observed in ANOVA were further analyzed using Tukey’s Honestly Significant Difference (HSD) post-hoc test to determine specific group differences. All analyses were performed using the IBM SPSS Statistics version 27.

### In-solution protein digestion

In total, 50 μg of proteins were resuspended in 50 μl of 50 mM ammonium bicarbonate (Sigma-Aldrich), heated to 80 C for 15 min, reduced with 2.5 μl of 100 mM (final concentration) for 30 mins at 60 C, and alkylated with 2.5 μl of 200 mM iodoacetamide (Sigma-Aldrich) at a final concentration of 20 mM at room temperature for 45 mins. Then the samples were digested with 2.0 μl (1.0 μg/μl) of Trypsin-LysC mixture enzyme (Mass Spec Grade, Promega) overnight at 37 C at a protein-to-enzyme ratio of 20:1 (w/w). The digested protein sample was desalted with 2.0 μl of concentrated trifluoroacetic acid (TFA, Sigma-Aldrich). After centrifugation at 14,000 rpm for 20 minutes, the supernatant was collected and submitted for proteomics analysis to the National Institute of Biotechnology Malaysia (NIBM).

### LC-MS/MS data analysis.

The data obtained from the mass spectrometer (Orbitrap Fusion, Thermo Fisher Scientific) were analyzed using Thermo Scientific^TM^ Proteome Discoverer^TM^ Software Version 2.1. Data were analyzed via the search engine Uniprot under taxonomy *Homo sapiens*, along with the contamination database provided by Thermo Scientific. Missed cleavage was set at 2; the selected fixed modification was carbamidomethyl (C). The false discovery rate (FDR) was set at less than 5%. Results generated by this software were filtered by the settings. All peptides were validated using the Percolator® algorithm, based on a q-value less than 5% FDR.

### Bioinformatics analysis

The raw data from mass spectrometry were analyzed using ShinyGO 0.77 (http://bioinformatics.sdstate.edu/go77/) to investigate the Gene Ontology (GO) component and KEGG pathway enrichment analysis. Pathways with a corrected p-value <0.05 were considered significant. Minimum p-values were adjusted using FDR. Pathways were selected based on their FDR value below 0.05 and fold enrichment above 5.0. Protein-protein interaction was analyzed using the STRING database (https://string-db.org/), followed by further protein classification for each protein cluster by Protein Analysis Through Evolutionary Relationships (PANTHER) pathway database (https://www.pantherdb.org/).

## Results

### Assessment of mesenchymal stem cells surface markers

**Figure 1:**
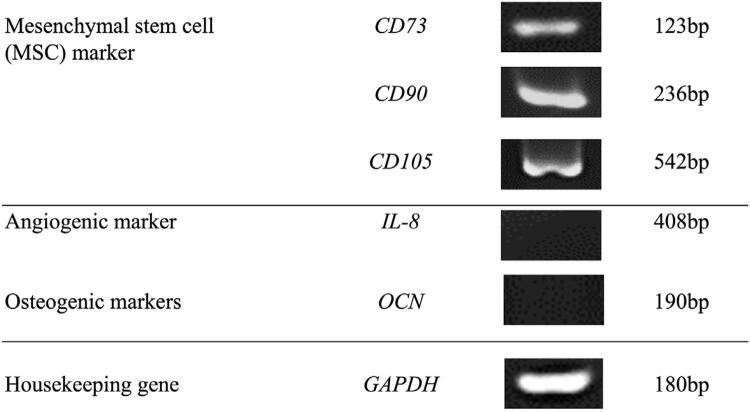
Mesenchymal stem cells surface markers of DPSC by PCR analysis. Incuded are MSC markers (CD73, CD90, CD105), and differentiated cell markers (IL-8 and OCN).

DPSC at early passages used in this study positively expressed *CD73*, *CD90*, and *CD105*, the common cell surface markers for mesenchymal stem cells. No expression of differentiation markers such as *IL-8* and *OCN* was observed (Figure 1).

### Collagen type 1 induce osteogenic differentiation of DPSC via different signalling pathways

**Figure 2:**
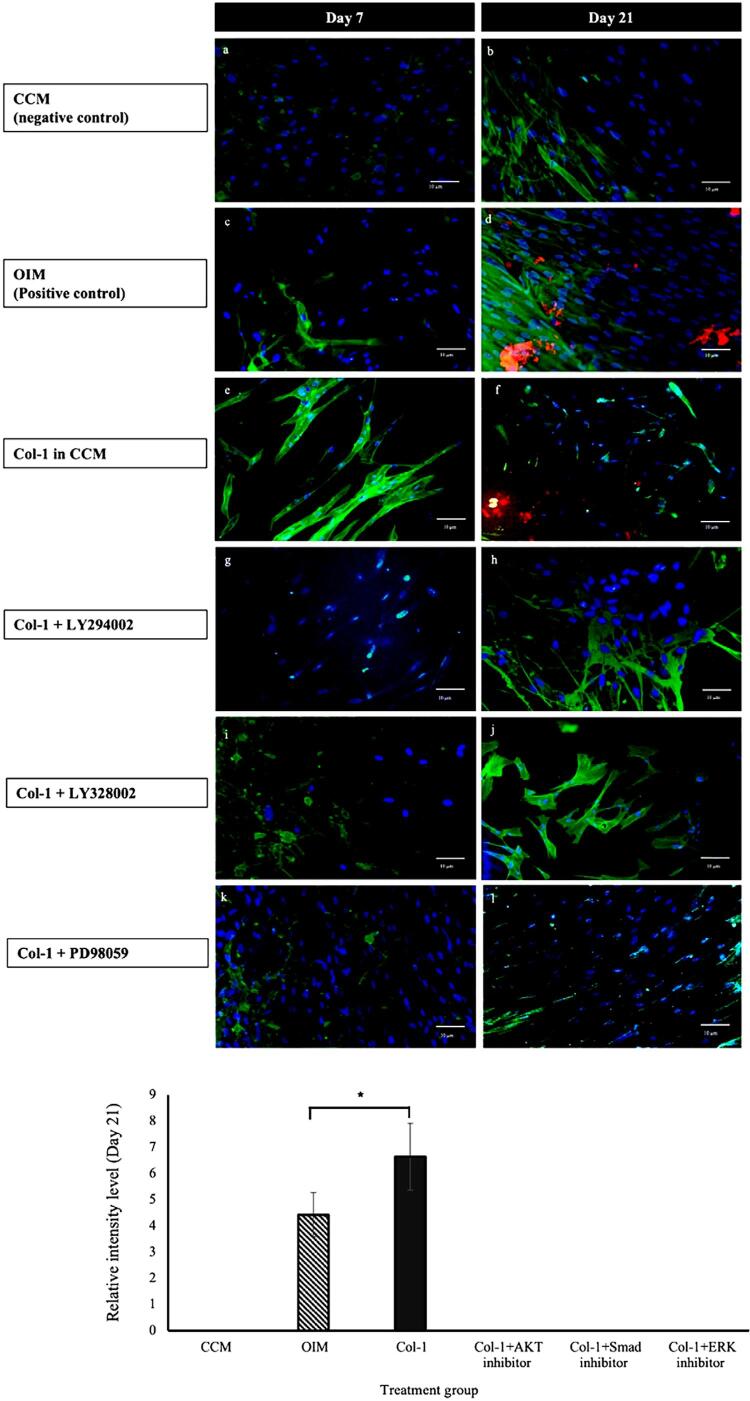
Immunocytochemistry analysis for the detection of OCN (red), cell nuclei (blue) and actin filament (green) in DPSC cultured in different condition on day 7 (a,c,e,g,i,k) and day 21 (b,d,f,h,j,l). (a,b) complete culture medium; (c,d) osteogenic induction medium; (c,f) Col-1 supplemented with complete culture medium, CCM; (g,h) supplemented with PI3K/AKT inhibitor; (i,j) supplemented with TGF-beta/Smad inhibitor; (k/l) supplemented with MAPK/ERK inhibitor.

OCN expression, indicative of higher bone turnover, was observed via immunocytochemistry analysis (Figure 2). All sample groups exhibited no expression of OCN on day 7 (a-f); however, after 21 days of induction in osteogenic medium or Col-1, the expression of OCN was found in both groups by positive staining of bone forming cells, as depicted by the intense red fluorescence staining (Figure 2h, i, l). The presence of a Col-1 scaffold showed alterations on the staining patterns of DPSC, with less visible staining observed on the actin filament and the cell nuclei, as seen in Figure 2i. Meanwhile, DPSC cultured with the MAPK/ERK inhibitor PD98059 showed slight staining of OCN, as depicted by an intermittent red fluorescence pattern (Figure 2l). Meanwhile, both DPSC groups cultured with the PI3K/AKT pathway inhibitor (LY294002) and the TGF-β/Smad pathway inhibitor (LY3200882) showed no staining of OCN in samples (Figure 2j, k), which suggested suppression of the signaling pathways associated with osteogenesis. Further comparison of staining intensity demonstrated significantly higher expression of OCN in Col-1 group compared to the positive control, the OIM group (p<0.05). Meanwhile, no expression of OCN was observed in the negative control group (CCM) to indicate the absence of osteogenesis within the samples.

### PI3K/AKT, TGF-β/Smad, and MAPK/ERK pathways involvement during stages of osteogenic differentiation in Col-1-induced DPSC

**Figure 3:**
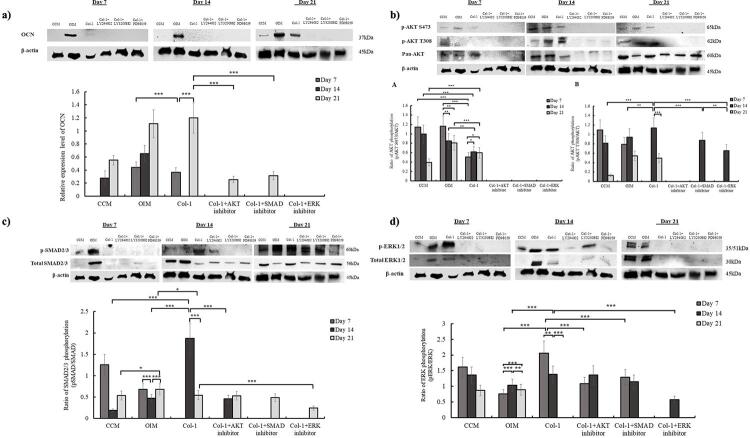
Western blot analysis in the expression of a) OCN (b) phospho-AKT at S473 and T308 (c) phospho-Smad2/3 and (d) phospho-ERK1/2 at day 7, 14 and 21 after DPSC was plated on Col-1, along with AKT, SMAD and ERK inhibitors. Phosphorylated ERK were normalised against total ERK. Each bar represents mean ± SEM (n=6). All p-values were based on two-way ANOVA, followed by Tukey test. Comparison between groups: *p<0.05, **p<0.01,***p<0.001.

The Western blot analysis of DPSC cultured on Col-1 displayed significantly lower expression of OCN compared to the OIM control group on day 7 (Figure 3a). Although OCN was absent on day 14, the expression was significantly elevated on day 21, demonstrating the role of Col-1 in modulating the osteogenic differentiation of DPSC, without the use of common osteogenic supplementation and induction medium. Further observation of the impact of inhibitors on Col-1 induction showed that PI3K/AKT and TGF-β/Smad inhibitors exhibited a significant reduction in OCN expression, whereas the MAPK/ERK inhibitor completely inhibited OCN expression on day 21 (Figure 3a). Together, the results demonstrated that PI3K/AKT, TGF-β/Smad, and MAPK/ERK pathways play an important role in Col-1-induced bone synthesis in the DPSC population.

Next, the expression of total protein and its phosphorylated form was observed to identify the key signaling pathway activated in Col-1-induced osteogenesis. DPSC cultured on Col-1 showed a constant expression of both phospho-AKT (p-AKT) S473 and T308 throughout 21 days of osteogenic differentiation (Figure 3b). Expression of phospho-AKT S473 significantly increased from day 7 to day 14 (*p*=0.02) and remained constant on day 21. Interestingly, the trend of p-AKT S473 expression in Col-1 increased over the 21-day experiment, as opposed to the control groups (both CCM and OIM), which showed a decline in expression. Moreover, the addition of PI3K/AKT, TGF-β/Smad, and MAPK/ERK inhibitors altogether completely diminished p-AKT S473 expression (Figure 3b).

Another site of AKT phosphorylation at T308 was also observed, and the data showed a different trend compared to site S473 (Figure 3b). DPSC cultured on Col-1 showed no p-AKT T308 activity on day 7, as opposed to the control groups (CCM and OIM). The phosphorylation of AKT at T308 peaked on day 14 in the Col-1 group and was significantly higher than the positive control OIM (*p*=0.003) (Figure 3b). However, by day 21, the expression of p-AKT T308 had declined (*p*<0.001) and showed no significant difference compared to the control OIM group. Treatment with the PI3K/AKT pathway inhibitor LY294002 completely inhibited p-AKT T308 activity throughout the 21-day experiment. Conversely, inhibition with TGF-β/Smad and MAPK/ERK inhibitors completely diminished the p-AKT T308 activity on day 7 and 21.

The activity of phospho-Smad2/3 (p-Smad2/3) was also analyzed to determine its role in Col-1-induced osteogenesis. Notably, p-Smad2/3 was normalized against total Smad activity, in which the expression of total protein in the Col-1 group differed from the control group (Figure 3c). DPSC cultured on the Col-1 scaffold exhibited no p-Smad2/3 activity on day 7 and peaked on day 14. Expression of p-Smad2/3 in the Col-1 group was significantly enhanced compared to the control OIM group (*p*<0.001). However, by day 21, p-Smad2/3 activity declined in the Col-1 group, with expression levels falling below those of the control OIM group (*p*=0.01). As for the inhibition assay, all pathway inhibitor groups exhibited no activity of p-Smad2/3 on day 7, similar to the control Col-1 control group, which also showed no expression. On day 14, p-Smad2/3 activity was completely inhibited by LY3200882 (TGF-β/Smad inhibitor) and PD98059 (MAPK/ERK inhibitor). However, inhibition by LY294002, the PI3K/AKT inhibitor, did not show a complete inhibition but did cause a significant decline in p-Smad2/3 expression levels (*p*<0.001). In contrast, on day 21, inhibition by PI3K/AKT and TGF-β/Smad inhibitors did not result in a significant reduction of p-Smad2/3 expression, whereas the MAPK/ERK inhibitor significantly suppressed the p-Smad2/3 expression.

Throughout the 21-day induction period, DPSC cultured on Col-1 exhibited a significantly higher expression of p-ERK activity on day 7 compared to the control OIM (*p<*0.001) (Figure 3d). However, p-ERK recorded a sharp decline from day 7 to 14 (*p=*0.002) and was completely absent on day 21, suggesting the involvement of the MAPK/ERK pathway at an early stage of osteogenesis. In addition, the pathway inhibition assay showed complete suppression of p-ERK1/2 when DPSC was cultured on Col-1 treated with PD98059 (MAPK/ERK pathway inhibitor) on day 7 and 21. Likewise, inhibition by PI3K/AKT and TGF-β/Smad pathway inhibitors significantly reduced the p-ERK activity on day 7 (all *p<*0.001), while activity was completely diminished on day 21 (similar to the Col-1 control). Inhibition assays performed on day 14 using PI3K/AKT and MAPK/ERK pathway inhibitors demonstrated downregulation of the p-ERK1/2 activity, whereas the use of TGF-β/Smad inhibitors, LY3200882 did not show any significant changes in the p-ERK1/2 expression, as compared to the Col-1 group without inhibitors.

### Functional enrichment analysis and KEGG pathway annotation

**Figure 4:**
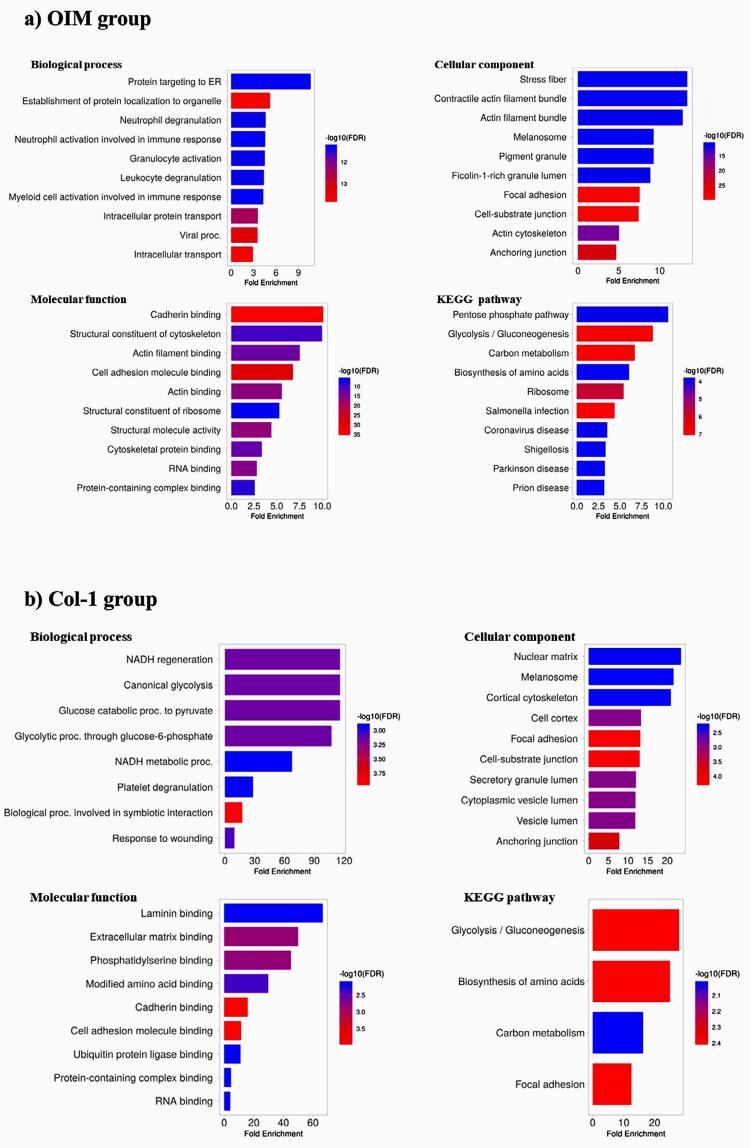
Functional enrichment analysis of gene ontology (GO) analysis and KEGG pathway enrichment for (a) OIM group and (b) Col-1 group.

Functional enrichment analysis on GO terms (biological processes, cellular components, and molecular functions) and KEGG pathway annotation were performed on day 21 samples. Analysis of the OIM group exhibited a biological process associated with the establishment of protein localization to organelles, with the lowest false discovery rate (FDR) of (FDR=6.7×10^-12^) and highest fold enrichment (>5) among the top 10 biological process (Figure 4a). As for the cellular components, the OIM group was found to be enriched in focal adhesion and cell-substrate junction, whereas the molecular functions were associated with cadherin binding and cell adhesion molecule binding. The gathered data revealed that the identified KEGG pathways with significant FDR observed for the OIM group were associated with glycolysis/gluconeogenesis, carbon metabolism, and Salmonella infection.

Meanwhile, the Col-1 group exhibited an association with biological processes involved in symbiotic interaction, NADH regeneration, and mainly the glycolysis process (Figure 4b). The cellular components in the group were also linked to focal adhesion and cell substrate junctions being the most significant. In addition, the significant molecular functions were associated with cadherin binding and cell adhesion molecule binding. Analysis also revealed that the identified KEGG pathways associated with the Col-1 group were mainly glycolysis/gluconeogenesis, amino acid biosynthesis, focal adhesion, and carbon metabolism.

**Figure 5:**
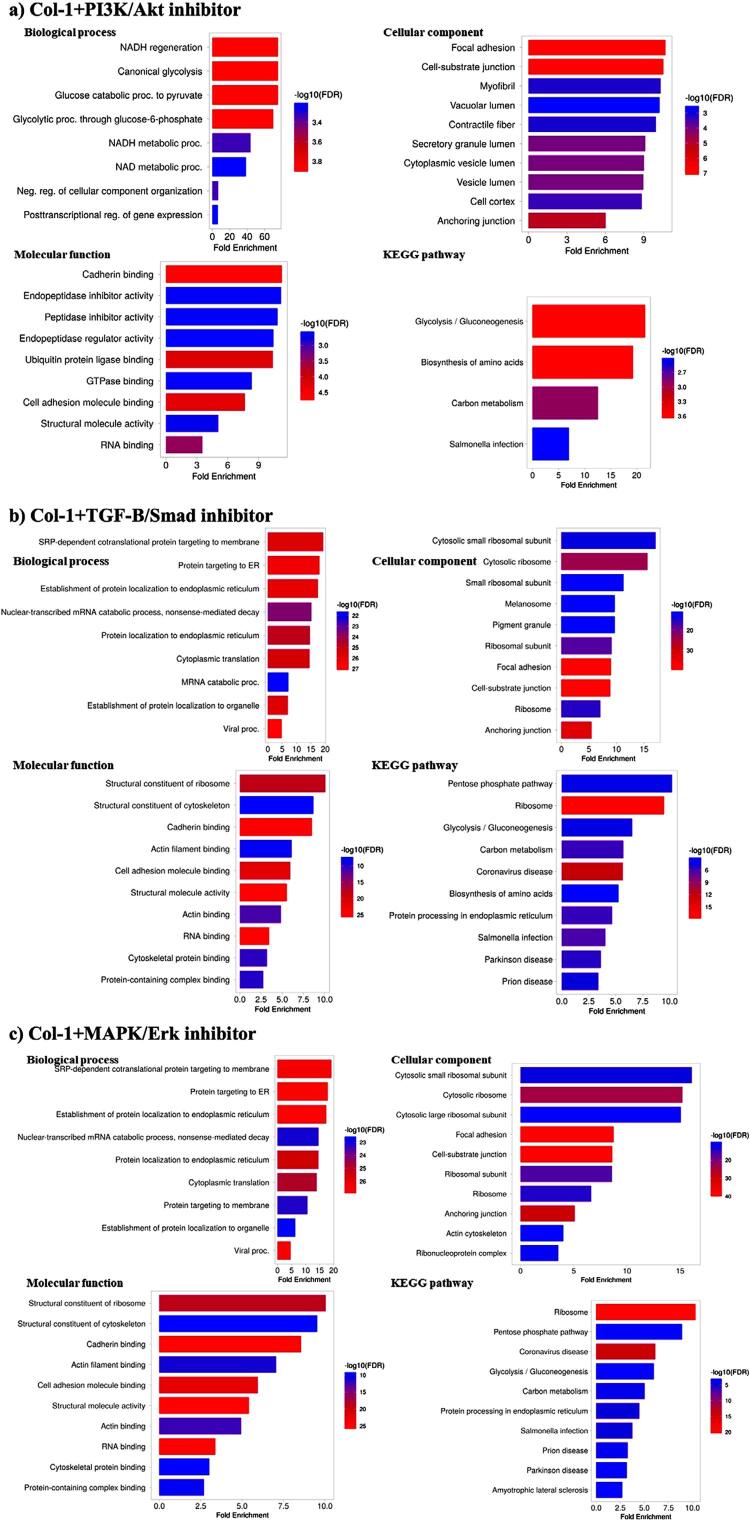
Functional enrichment analysis of gene ontology (GO) analysis and KEGG pathway enrichment for (a) Col-1+PI3K/AKT inhibitor, (b) Col-1+TGF-β/Smad inhibitor and (c) Col-1+MAPK/ERK inhibitor

Further on, the functional enrichment analysis was performed to observe the effect of signaling inhibitors on the cellular functions and annotated pathways (Figure 5). The analysis of cellular components demonstrated that the Col-1+PI3K/AKT inhibitor group showed an association with NADH regeneration, particularly the glycolysis process elements. Meanwhile the biological process data revealed a biological activity linked to focal adhesion, with the cell-substrate junction being the most significant. The molecular functions of the group also exhibited cadherin binding, ubiquitin protein ligase binding, and cell adhesion molecule binding. The Col-1+PI3K/AKT inhibitor group showed lower fold enrichment and higher FDR compared to Col-1 group, whereas the KEGG pathway annotations were considerably similar. The similar pathways were glycolysis/gluconeogenesis, amino acid biosynthesis, and carbon metabolism (Figure 5a).

The functional enrichment analysis of the Col-1+TGF-β/Smad inhibitor group was distinct compared to that of the Col-1 group. The biological processes of the group were mainly related to endoplasmic reticulum (ER) translocation. As for cellular components, focal adhesion and cell-substrate junction were the most significant elements, yet cytosolic ribosome elements exhibited the top three highest fold enrichment values (>15). The molecular functions were linked to cadherin binding, structural molecule binding, RNA binding, and cell adhesion molecule binding with some of the most significant FDR value (FDR<0.001). KEGG pathway annotation indicated that the proteins were mostly associated with the pentose phosphate pathway, ribosome, and glycolysis/gluconeogenesis, based on fold enrichment values (≥7) (Figure 5b).

On the other hand, the functional enrichment analysis for the Col-1+MAPK/ERK inhibitor group was found relatively consistent with that of the Col-1+TGF-β/Smad inhibitor group (Figure 5c). The top three biological processes with the most significant FDR values (FDR<0.001) involved endoplasmic reticulum-related elements. Similar to the previous group, the most significant cellular components were focal adhesion and cell-substrate junction, with slightly lower fold enrichment. The molecular functions of the group were mainly associated with cadherin binding, structural molecule activity, and cell adhesion molecule binding. KEGG pathway annotations further revealed a highly enriched association with the ribosome pathway, followed by the pentose phosphate pathway.

### Protein-protein interaction (PPI) network and osteogenic related proteins in the Col-1-induced osteogenesis group

Protein profiling analysis identified 42 groups of protein functions expressed in the Col-1 group after 21 days of osteoinduction. However, only 16 proteins were interconnected in PPI network, with six unclustered proteins, the rest were unidentified. The unclustered proteins included parathymosin (PTM, accession number: P20962), inter-alpha-trypsin inhibitor heavy chain H3 (ITIH3, accession number: Q06033), protein CDV3 homolog (CDV3, accession number: Q9UKY7), cytochrome b5 type B (CYB5B, accession number: O43169), cilia-and-flagella-associated protein 100 (CFAP100, accession number: Q494V2), and C-type lectin domain family 11 member A (CLEC11A, accession number: Q9Y240).

**Figure 6:**
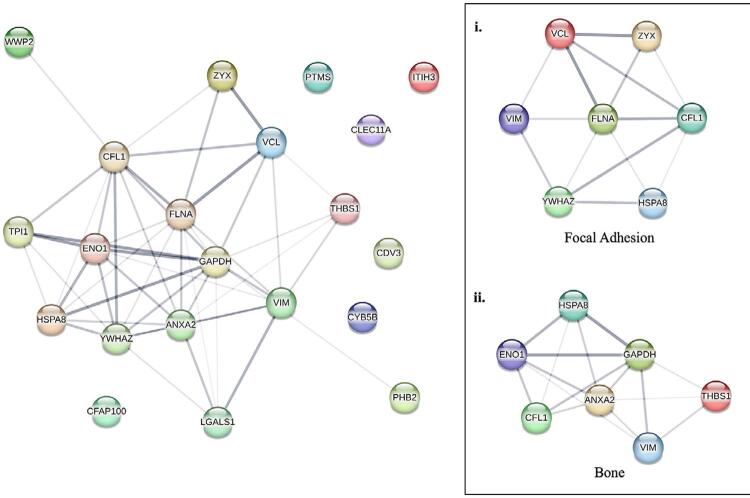
Protein-protein interaction network of Col-1 group. (i) Interconnected protein that involved in focal adhesion mechanism of Col-1 and (ii) protein component involved in bone formation in Col-1 group after 21 days of osteoinduction.

**Figure 7:**
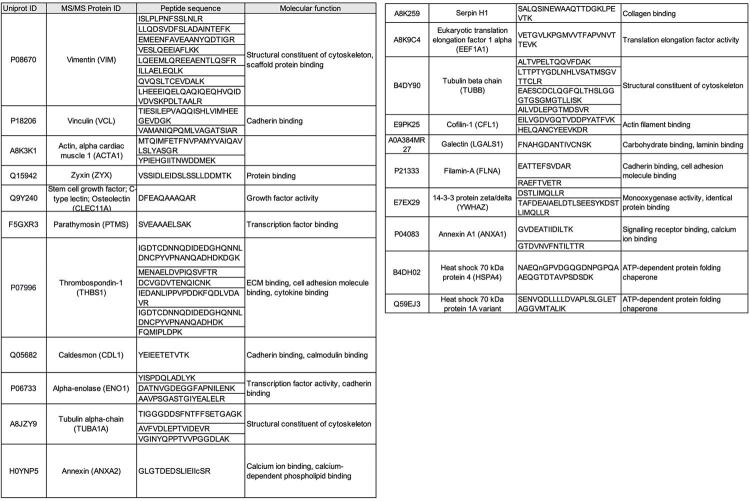
The identified proteins related to osteogenic differentiation in Col-1 induced DPSC.

Within the interconnected PPI network, seven proteins were found to be involved and relevant to the focal adhesion mechanism and bone formation (Figure 6). Proteins associated with focal adhesion mechanism were Vimentin (VIM), Vinculin (VCL), Zyxin (ZYX), Filamin-A (FLNA), 14-3-3 protein zeta/delta (YWHAZ), Cofilin 1 (CFL1), and Heat Shock 70kDa Protein 8 (HSPA8), whereas proteins involved in Col-1 bone formation included HSPA8, Enolase 1 (ENO1), GAPDH, CFL1, Annexin A2 (ANXA2), VIM, and Thrombospondin 1 (THBS1). The role of Col-1 in promoting osteogenic differentiation of DPSC was supported by the expression of osteogenic related proteins identified within the sample. Overall, 21 proteins were identified as playing a vital role in the osteoinduction process (Figure 7).

### Col-1 enhanced the regulation of cellular functions

**Figure 8:**
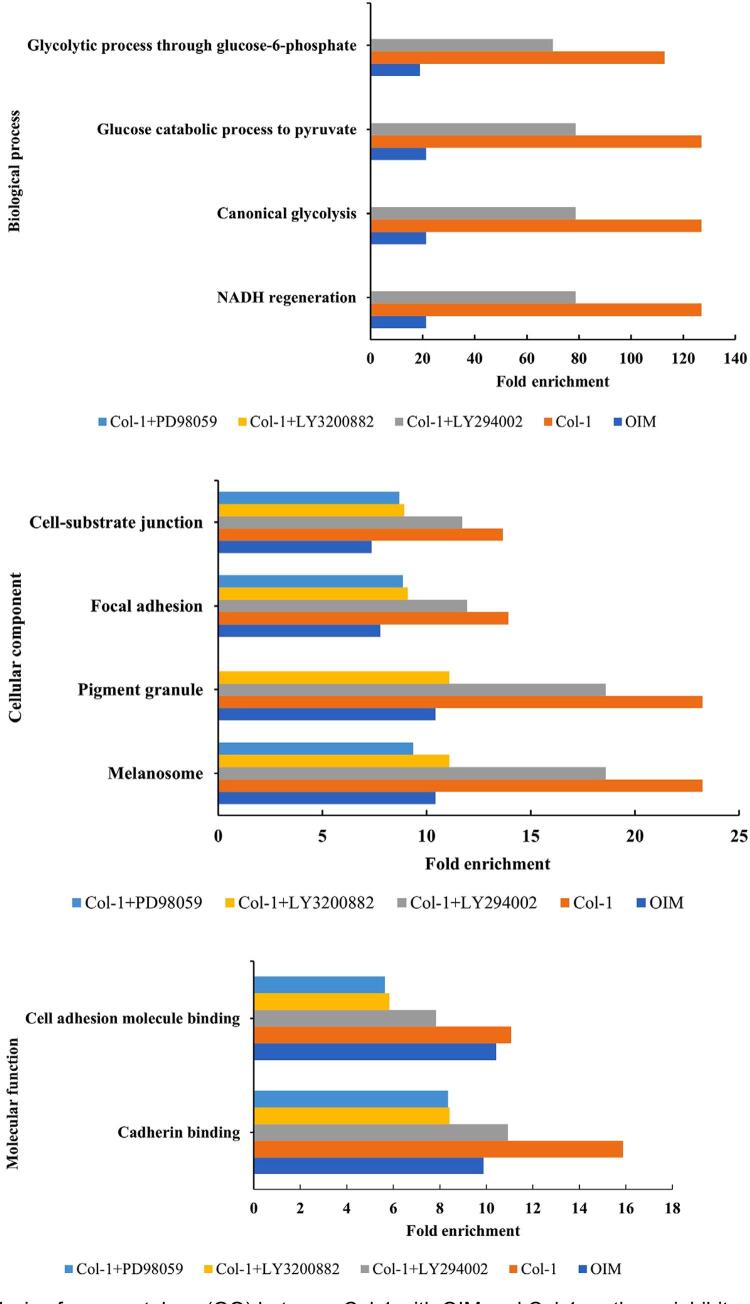
Comparative analysis of gene ontology (GO) between Col-1 with OIM and Col-1+pathway inhibitors.

Comparative analysis of GO components revealed that Col-1 enhanced most aspects of cellular regulation—specifically glycolysis in the biological processes, cell-substrate junction and focal adhesion in cellular components, and binding activity function (Figure 8). Inhibition of the PI3K/AKT, TGF-β/Smad, and MAPK/ERK signaling pathways led to a decrease in fold enrichment compared to the control Col-1 group. Although inhibition of the PI3K/AKT pathway decreased fold enrichment in most aspects of cellular regulation, inhibition of TGF-β/Smad and MAPK/ERK resulted in the cellular regulation focusing on endoplasmic reticulum related proteins (Figure 5b,4c).

## Discussion

DPSCs offer a promising alternative for bone tissue regeneration due to its stemness properties. Positive expression of MSC surface markers is one of the hallmarks of stemness, which was analyzed via PCR due to its simplicity, sensitivity, and rapid determination of the gene expression profile.^10^ DPSC demonstrated positive expression of *CD73*, *CD90*, and *CD105*, in line with several previous studies employing dental stem cells.^10,11^ Moreover, the DPSC demonstrated negative expression of the angiogenic marker *IL-8* and the osteogenic marker *OCN*, indicating the cells remained undifferentiated.

Collagen type 1 is the main component of the bone extracellular matrix (ECM), making it a promising scaffold material for bone tissue engineering. This study demonstrated the capacity of Col-1 to induce the osteogenic differentiation of DPSC, independent of typical osteogenic supplementation consisting of ascorbic acid 2-phosphate, β-glycerophosphate, and dexamethasone. This is consistent with previous studies showing that collagen provides an optimal cellular environment for cells to respond to differentiation signals.^12,13^

The current study employs OCN as a bone formation biomarker due to its specificity and sensitivity. OCN is regarded as a late-stage bone marker in the osteogenic differentiation phase due to its production by cells responsible for mineralization, such as osteoblasts. Positive osteocalcin expression and localization within the cells were confirmed via immunocytochemistry and Western blot analysis. The results also revealed that OCN protein expression in Col-1 group peaked only on day 21, which aligns with several other *in vitro* studies on OCN expression induced by mediators other than osteogenic induction media.^14,15^ Altogether, the data suggested an active role and involvement of Col-1 in the later stages of DPSC osteogenic differentiation. It is worth noting that OCN production, which results from osteogenic differentiation, can vary depending on the biomaterials and types of MSCs used. For instance, different types of Col-1 scaffolds consisting of different compositions can impact the level of osteogenic differentiation and OCN production. A previous study using a hydroxylapatite-collagen hybrid scaffold on hBMSC demonstrated an elevated OCN level throughout 21 days of osteogenesis.^16^ Additionally, a different study using hMSCs on pure Col-1 scaffold and a modified Col-1 with complexed calcium polyphosphate microparticles showed that OCN was not expressed on day 21 but was detectable by day 35.^17^ These findings suggested that increased OCN production marks the final stage of osteogenic differentiation (mineralization), as observed in most osteogenesis studies conducted within 21 days of differentiation.

Further investigation was performed to identify the predominant signaling pathway responsible for the osteoinductive effect of Col-1, via pathway inhibition assays and observation of phosphorylated protein activity. The inhibition by LY294002 (PI3K/AKT inhibitor), LY3200882 (TGF-β/Smad inhibitor), and PD98059 (MAPK/ERK inhibitor) led to reduced expression of OCN throughout the 21-day osteoinduction period. Similarly, this result aligns with data from previous studies, which postulated the involvement of PI3K/AKT, TGF-β/Smad, and MAPK/ERK during MSC osteogenesis when each pathway was studied independently.^18,19^ While osteogenic induction media resulted in relatively stable expression of phosphorylated AKT, Smad, and ERK throughout the 21-day induction period, Col-1 application demonstrated stage-specific activation of these pathways.

**Figure 9:**
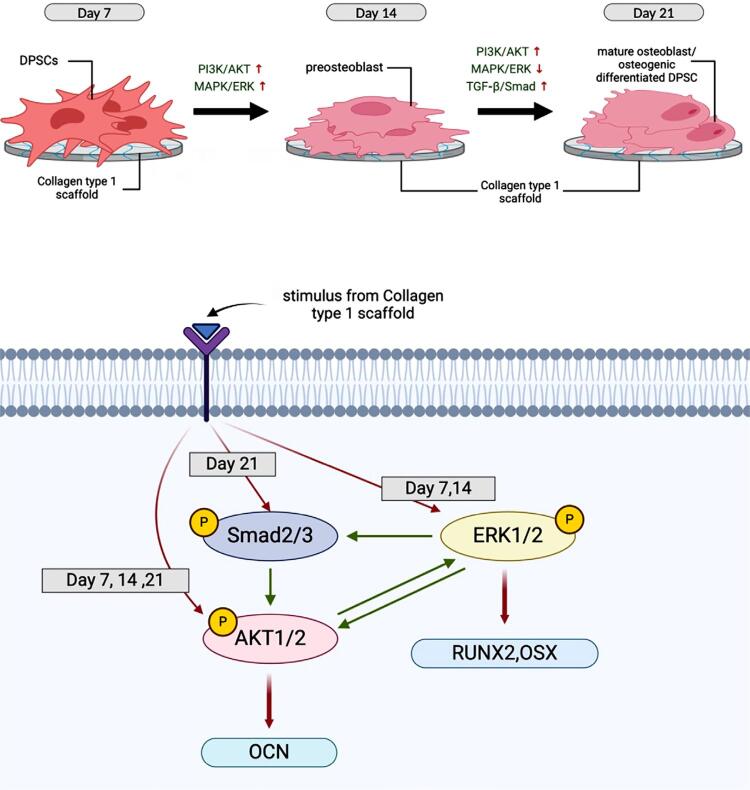
Summary of the signalling mechanisms of Col-1 osteogenic differentiation in DPSC.

The PI3K/AKT signaling pathway plays an important role in various aspects of cellular regulation, including cell growth, proliferation, and differentiation.^20^ Phosphorylation of AKT at both S473 and T308 indicates the active role of PI3K/AKT pathway in the osteogenic differentiation of DPSC.^21^ Additionally, inhibition by LY294002 (PI3K/AKT inhibitor), LY3200882 (TGF-β/Smad inhibitor), and PD98059 (MAPK/ERK inhibitor) suppressed phosphorylation at both S473 and T308, suggesting a possible crosstalk between these pathways, as suggested in Figure 9. Crosstalk between PI3K/AKT and TGF-β/Smad has been reported in previous studies, revealing the dependency of PI3K/AKT pathway on Smad3 expression and the involvement of p38 MAPK pathway throughout the process.^24,25^

Although PI3K/AKT pathway activity was influenced by Smad2/3 activity, Smad2/3 regulation was found to be independent of PI3K/AKT activity, as a study demonstrated that the translocation of Smad2/3 was not inhibited by PI3K/AKT.^26^ The inhibition of AKT activity by PD98059 observed in this study contrasts with data published on hMSC osteogenesis using collagen 1 coating, in which PD98059 did not reduce the expression of AKT.^27^ These discrepancies could be attributed to differences in pathway interactions, likely due to the various stem cell types employed in each study. In this regard, further research into the types of stem cells employed for osteogenic with these pathway inhibitors could aid in elucidating the dynamics of the cells, stimuli, and pathway interactions.

In our investigation, the MAPK/ERK pathway mechanism in Col-1-induced osteogenic differentiation was found to be stage-dependent, specifically active during the early stage. This finding aligns with a similar study that reported a significant increase of ERK activity from days 5 to 10, followed by a decrease afterwards.^28,29^ Interestingly, ERK activity was inhibited by LY294002 (PI3K/AKT inhibitor), which aligns with previously published data.^27^ Thus, it is postulated that the PI3K/AKT inhibitor may interact with the ERK pathway by binding to the ATP binding site of the MAPK molecules.^30^

The TGF-β/Smad pathway is known for its role in bone formation during the early stages of bone cells differentiation and for inhibiting the process at later stages.^31^ However, our findings showed that Smad2/3 activity peaked during the later stages of osteogenic differentiation. This result suggests that TGF-β/Smad may also be regulated by another signaling pathway during the process, such as PI3K/AKT.^32,33^ Treatment with LY3200882 (a TGF-β/Smad inhibitor) suppressed AKT activity, indicating that TGF-β/Smad acts as an upstream regulator. This effect was mediated by PI3K/AKT during the later stages of osteogenic differentiation. Likewise, TGF-β/Smad activity was also suppressed when treated with PD98059 (MAPK/ERK inhibitor) on day 21, suggesting that Smad2/3 may also depend on the MAPK/ERK pathway. The overall signaling dynamics during the later stages of osteogenic differentiation suggest an ERK-activated, PI3K/AKT-mediated TGF-β signaling complex.

Notably, Col-1-induced osteogenic differentiation showed no expression of OCN on day 14, despite the positive expression of p-AKT, p-Smad2/3, and p-ERK1/2 in the Col-1 scaffold group. This could be due to the temporal dynamics and feedback mechanisms of the signaling pathway involved in the process. Our study recorded the involvement of all signaling pathway activities on day 14 of osteogenic differentiation. The TGF-β/Smad and ERK pathways, specifically, were found to inhibit osteogenic differentiation at this later stage.^31,34^

Proteomic analysis further suggested that the Col-1 scaffold enhanced most of the gene ontology (GO) component, specifically the energy production in the form of ATP via glycolysis and NADH regeneration, as well as cell binding and focal adhesion. Osteogenic differentiation is an energy-consuming process supported by high metabolic activities such as glycolysis and NADH regeneration, particularly among mature osteoblasts.^35,36^ In addition to cellular metabolic activities, KEGG pathway annotation and the interconnected PPI network also indicated the involvement of most proteins in focal adhesion activities. Focal adhesion is critical in osteoblast mechano-transduction, converting mechanical stress into biochemical signals and incorporating them into cellular activities.^37^ Theoretically, ZYX and VCL may modulate osteogenic differentiation by providing mechanical regulation and facilitating cell-cell and cell-matrix adhesion.^38,39^

Another focal adhesion protein, 14-3-3 protein zeta/delta (14-3-3ζ, YWHAZ), is classified as a scaffold/adaptor protein. YWHAZ modulates multiple cell signaling transduction via phosphoserine protein binding.^40^ Previous research also revealed the role of YWHAZ in cytoskeletal structure regulation.^41^ This regulation occurs via interactions with phosphorylated cofilin (CFL1) and the upstream regulators.^42^ These interactions contribute to the conservation of phosphorylated and stabilized actin filaments. In addition, YWHAZ was found to be involved in the phosphorylation of AKT signaling, which upregulates the stability of *Cbfa1/Runx2* protein for osteogenic lineage.^43^

It is worth noting that unclustered proteins such as C-type lectin domain family 11 member A (CLEC11A/Osteolectin) may also contribute to Col-1-induced osteogenic differentiation of DPSC. CLEC11A is a potent bone anabolic factor known to enhance bone density in both *in vitro* and *in vivo* models by stimulating the differentiation of mesenchymal progenitors into mature osteoblasts.^44^ Proteins such as ENO1, found in the bone formation cluster, are also involved in cellular metabolic processes, including glycolysis.^45^ ENO1 is also associated with the regulation of stemness in certain cells types by improving the glycolytic process.^46^

## Conclusion

Collectively, this study suggested that PI3K/AKT plays an active role and is predominantly involved in mediating the osteogenic differentiation of DPSC on a Col-1 scaffold via the focal adhesion pathway. This study also found involvement of MAPK/ERK and TGF-β/Smad pathways at different stages of differentiation, mediated by PI3K/AKT. Moreover, bioinformatic analysis of Col-1-induced DPSC also revealed that majority of identified proteins are associated with glycolysis activity, providing sufficient energy for the process, and with the focal adhesion mechanism from the environmental stimuli of cell binding to the collagen scaffold.
